# Association between white matter microstructural and functional abnormalities and clinical characteristics in migraine without aura: a mediation analysis

**DOI:** 10.3389/fneur.2025.1693789

**Published:** 2025-11-12

**Authors:** Qixuan Fu, Chaorong Xie, Yangxu Ou, Zhiyang Zhang, Xuhong Yang, Xiangdong Luo, Qinyi Yan, Tong Wang, Xiao Wang, Ling Zhao

**Affiliations:** 1 School of Acupuncture Moxibustion and Tuina, Chengdu University of Traditional Chinese Medicine, Chengdu, Sichuan, China; 2 Department of Neurology, Hospital of Chengdu University of Traditional Chinese Medicine, Chengdu, Sichuan, China; 3 Department of Neurology, Sichuan Provincial People's Hospital, Chengdu, Sichuan, China; 4 Acupoint Effect Key Laboratory of Sichuan Province, Chengdu, Sichuan, China

**Keywords:** amplitude of low-frequency fluctuation, degree centrality, pain, tract-based spatialstatistics, white matter tracts

## Abstract

**Background:**

Migraine without aura (MWoA) is a neurological disorder associated with structural and functional abnormalities in white matter (WM). However, interactions between clinical characteristics and WM abnormalities of microstructure and function in MWoA have remained underexplored. In this study, we aimed to investigate these associations and broaden the understanding of the pathophysiology of MWoA.

**Methods:**

A total of 51 MWoA patients and 51 healthy controls (HCs) underwent magnetic resonance imaging (MRI). Microstructural WM abnormalities were assessed using tract-based spatial statistics (TBSS). Functional alterations were evaluated by measuring the amplitude of low-frequency fluctuation (ALFF) and degree centrality (DC). Spearman’s rank correlation was used to assess the association between these abnormalities and clinical characteristics such as frequency, intensity, and disease progression of MWoA. We also conducted a region-level functional connectivity analysis, followed by mediation analysis to explore potential pathways linking WM abnormalities to clinical characteristics.

**Results:**

This study showed that, compared to HCs, MWoA patients showed decreased fractional anisotropy (FA), axial dispersion (AD), and DC and increased ALFF in the left frontopontine (FPT_L), decreased FA and AD in the forceps major (CC_ForcepsMajor), and decreased ALFF in the forceps minor (CC_ForcepsMinor). Among them, ALFF in the CC_ForcepsMinor and DC, FA, and AD in the FPT_L were inversely correlated with disease duration (*p* < 0.05). FA and AD in the CC_ForcepsMajor were inversely correlated with visual analog scale (VAS) scores (*p* < 0.05). Exploratory mediation analysis suggested that functional and microstructural abnormalities in the corpus callosum (CC) subregions may mediate the relationship between the DC value in the FPT-L and disease duration among MWoA patients.

**Conclusion:**

This study reveals concomitant alterations in the function and microstructure of WM in the CC subregions and the FPT_L among MWoA patients. These alterations are significantly correlated with clinical characteristics and suggest that these abnormalities in functional fluctuations and WM integrity may serve as mediators between reduced network integration and disease duration in MWoA. These findings support the WM abnormality hypothesis and deepen our understanding of the pathophysiological mechanisms underlying MWoA.

## 1 Introduction

Migraine without aura (MWoA) is a highly prevalent and disabling neurological disorder that often follows a chronic course (1–3). It is characterized by dysregulation of the central nervous system, leading to clinical symptoms such as pain and sensory hypersensitivity (4). Current research has identified abnormalities in both brain structure and function in MWoA that correlate with clinical characteristics (5–7). However, resting-state functional magnetic resonance imaging (rs-fMRI) studies in MWoA have predominantly focused on gray matter (GM), overlooking the synergistic roles of white matter (WM) function and microstructure (5, 8, 9). Previous investigations on WM abnormalities in MWoA relied heavily on diffusion tensor imaging (DTI) for structural characterization. Several studies have demonstrated decreased WM fiber tract integrity in MWoA, primarily showing significantly reduced fractional anisotropy (FA) in pain-related brain regions such as the cerebellum, brainstem, corpus callosum (CC), and thalamus. Moreover, these reductions in FA have been correlated with disease duration and headache frequency (10–12). Another study found no significant microstructural changes in the WM of MWoA patients (13), leading to inconsistent conclusions. Additionally, a study on WM function confirmed changes associated with MWoA, but the pattern of functional abnormalities remains unclear (14).

Current research has established that WM microstructural and functional abnormalities are stable neuropathological signatures (15, 16). Their co-varying patterns are believed to accurately reflect disease mechanisms. For instance, during the preclinical stage of Alzheimer’s disease, concurrent functional and structural alterations in specific regions serve as early biomarkers predicting disease progression (17). Similarly, schizophrenia studies demonstrate that WM tract abnormalities correlate significantly with psychotic symptoms and disease progression (18). Collectively, these findings provide novel insights into the pathophysiological underpinnings of neurological disorders. They affirm the value of multimodal WM integration analysis, which transcends the constraints of unimodal approaches by combining structural and functional dimensions to elucidate the relationship between WM abnormalities and clinical symptoms. The triadic relationship among WM function, WM microstructure, and clinical characteristics in MWoA needs further exploration. Consequently, applying a multiscale WM analysis to MWoA will provide novel perspectives for deciphering its neuropathological mechanisms.

In this study, we integrate DTI and rs-fMRI, combining tract-based spatial statistics (TBSS) analysis, amplitude of low-frequency fluctuation (ALFF), and degree centrality (DC), to assess WM microstructural integrity and the abnormalities of fluctuations and integration (19), along with their associations with clinical characteristics. Crucially, this study is the first to apply mediation analysis to WM research in MWoA, aiming to elucidate the intricate association between WM microstructure, WM function, and clinical characteristics. We investigated potential mediating relationships among these variables through a theoretical model to uncover possible indirect pathways. This analytical approach integrates multimodal neuroimaging data, overcoming limitations inherent in previous single-modality perspectives. We hypothesize that co-occurring WM microstructural and functional abnormalities in specific brain regions collectively drive clinical characteristics in MWoA through mediation pathways.

## 2 Materials and methods

### 2.1 Participants

This cross-sectional study initially enrolled 105 participants. MWoA patients (N = 51) were recruited from the Neurology Outpatient Department of the Hospital of Chengdu University of Traditional Chinese Medicine and Sichuan Provincial People’s Hospital between February 2022 and December 2024. Healthy controls (HCs) were concurrently recruited (*N* = 51). The study was approved by the local ethics committee (authority number: 2020KL-003), registered with the China Clinical Trials Registry (registration number: ChiCTR2000032308), and written informed consent was obtained from all participants.

The diagnosis of MWoA was established according to the criteria outlined in the International Classification of Headache Disorders, 3rd Edition (ICHD-3) (20). The inclusion criteria for MWoA patients were as follows: (1) age between 18 and 55 years, with migraine onset at or before 50 years of age; (2) fulfillment of the diagnostic criteria specified in the ICHD-3; (3) having experienced between 2 and 15 headache episodes within a 4-week period during the preceding 3 months; (4) having baseline headache pain intensity classified as moderate, with visual analog scale (VAS) scores ranging from 3 to 7; (5) illness duration exceeding 1 year; and (6) willingness and ability to maintain a baseline headache diary.

HCs were required to meet the following criteria: (1) age between 18 and 55 years; (2) right-handed; and (3) no significant health issues, based on medical history and routine physical examination.

The exclusion criteria for all participants were as follows: (1) diagnosed with other types of primary headaches; (2) significant comorbid medical conditions, including cardiovascular, cerebrovascular, hepatic, renal, hematopoietic, or other systemic organic diseases; (3) diagnosed with sleep disorders, major psychiatric disorders (such as depression or anxiety disorders), or other neurological diseases; (4) a history of significant head trauma, or the presence of neuropsychiatric or intellectual disorders that could impair the ability to provide reliable questionnaire data; (5) pregnancy, lactation, or attempted conception within the past six months; (6) alcohol or drug abuse; (7) receipt of acupuncture or other preventive treatments within the preceding 4 weeks; (8) general contraindications for MRI, including metallic implants or claustrophobia; (9) severe cranial anatomical asymmetry or identifiable brain lesions on MRI; (10) inability to understand or record a headache diary; and (11) participation in similar research studies within the past 3 months.

### 2.2 Clinical characteristic evaluation

Demographic data (age and sex) were recorded for all participants. In addition, we evaluated the headache intensity and frequency based on the headache diaries during the observation period. To minimize potential confounding from acute medications, a protocol was implemented that restricted rescue analgesia to ibuprofen only. All use of rescue medication was prospectively documented in headache diaries. Specific clinical indicators assessed were as follows: (1) disease duration (months); (2) number of migraine days per month; (3) duration of each migraine (hours); (4) VAS score, where “0” indicates no pain and “10” indicates the most intense pain imaginable; (5) Headache Impact Test (HIT-6) scores; and (6) Migraine-Specific Quality-of-Life Questionnaire (MSQ) scores.

### 2.3 MRI acquisition

All patients diagnosed with MWoA underwent MRI during the interictal period, indicating that all patients were in an attack-free state 3 days before, on the day of, and 3 days after the MRI scan (21). MRI data were collected using a 3 T MRI scanner (Allegra, Siemens Medical System, Erlangen, Germany), equipped with a high-speed gradient coil, which was located in the Hospital of Chengdu University of Traditional Chinese Medicine. High-resolution three-dimensional T1-weighted structural images were acquired using a fast spoiled gradient-echo (FSPGR) (22) sequence with the following parameters (51 MWoA and 51 HCs): repetition time (TR)/echo time (TE) = 2,530/3.4 ms, matrix = 512 × 512, flip angle = 12°, and slice thickness = 1 mm. Diffusion-weighted images were acquired using a single-shot spin-echo EPI sequence with the following parameters: TR/TE = 8,400/91 ms, field of view (FOV) = 256 × 256 mm^2^, matrix = 128 × 128, 64 slices with a thickness of 3 mm, and a diffusion weighting scheme of 30 non-collinear directions at b = 1,000 s/mm^2^, along with one additional volume acquired at b = 0 s/mm^2^. The rs-fMRI images were collected using a gradient echo sequence with the following parameters: 30 continuous slices with a slice thickness of 5 mm, TR/TE of 2,000/30 ms, flip angle = 90°, FOV = 240 × 240 mm^2^, matrix = 64 × 64, and voxel size = 3.75 × 3.75 × 5 mm^3^. All participants were instructed to rest with their eyes closed, to avoid thinking of anything in particular, and to refrain from falling asleep during the rs-fMRI scan.

### 2.4 Image processing

In the DTI preprocessing pipeline, the following steps were performed for each participant using the FMRIB software library (FSL v6.0.7.12, https://fsl.fmrib.ox.ac.uk/fsl). First, the brain extraction tool was applied to the initial non-diffusion weighted volume to generate a binary brain mask. Next, the FSL eddy current tool was used to correct for eddy current distortions and motion artifacts (23). Finally, a diffusion tensor model was fitted to each voxel using a conventional linear least squares method implemented in DTIFIT within FMRIB’s Diffusion Toolbox.

Rs-fMRI data preprocessing was performed using Statistical Parametric Mapping software (SPM12; http://www.fil.ion.ucl.ac.uk/spm/software/spm12) and MATLAB (The MathWorks, Natick, MA, USA) (24). For each participant, the first five volumes were discarded to allow for magnetic field stabilization and adaptation to the scanning environment. The remaining images underwent slice timing correction to account for differences in acquisition time across slices, followed by realignment to the first volume using a six-parameter rigid body transformation to correct for head motion. The resulting mean functional image was then co-registered to the high-resolution T1-weighted structural image. T1 images were segmented to generate tissue probability maps for GM, WM, and cerebrospinal fluid (CSF), along with the corresponding deformation fields. These deformation fields were applied to spatially normalize all functional images to the standard Montreal Neurological Institute (MNI) space, with resampling to 3 × 3 × 3 mm^3^ isotropic voxels. Subsequently, temporal preprocessing was performed. Nuisance covariates were regressed out using a general linear model, linear trends were removed, and a temporal band-pass filter (0.01–0.08 Hz) was applied to isolate low-frequency fluctuations. Finally, spatial smoothing was conducted using a Gaussian kernel with a full-width at half-maximum (FWHM) of 6 mm.

### 2.5 TBSS analysis

Further analyses were performed using TBSS (25). First, the fraction anisotropy (FA), mean diffusivity (MD), radial diffusivity (RD), and axial diffusion (AD) images of all participants were non-linearly aligned to the mean FA template vs. non-FA template from the IIT Human Brain Atlas (www.nitrc.org/projects/iit) using FNIRT. The IIT Human Brain Atlas was chosen because it is derived from a high-quality, contemporary DTI template, offering more precise spatial normalization and probabilistic WM tract definitions (26). Second, each subject’s four metric images were projected onto the skeleton image to generate a merged image. Third, the skeleton images were thresholded to FA > 0.2 to retain only the major tracts. Fourth, each subject’s aligned data were projected onto this skeleton. Finally, group comparisons of FA, MD, RD, and AD were conducted between MWoA and HCs. For details regarding the statistical methods, see Section 2.8.1.

### 2.6 Regionconnect analysis

To extract standardized connectivity information for abnormal WM regions, we utilized the Regionconnect method. Leveraging connectivity-based multi-layer labels, Regionconnect rapidly accesses and integrates the IIT atlas-aligned healthy young adult connectome (27). The most probable WM connection in each IIT Human Brain Atlas voxel were visualized using FSLeyes.

### 2.7 ALFF and DC analysis

Dynamic rs-fMRI metrics were analyzed using a toolbox for Data Processing & Analysis for Brain Imaging (DPABI V8.2, https://rfmri.org/DPABI) (28). The ALFF was computed as follows: For each voxel, the preprocessed time series was converted to a power spectrum using a Fast Fourier transform (FFT); the ALFF value was then derived by averaging the square root of the power spectrum across the 0.01–0.08 Hz frequency band (29); and finally, every voxel’s ALFF value was normalized by the global mean ALFF value of the individual brain. The BOLD signal in WM is of lower amplitude and higher in physiological noise (30). The ALFF is sensitive to global noise, thereby enabling more sensitive detection of potentially spontaneous neural activity in WM, albeit with a potentially greater inclusion of physiological noise (31). Furthermore, ALFF has been widely adopted as the primary metric in existing WM functional magnetic resonance studies (32, 33). Our choice of ALFF ensures that our findings maintain interpretability with existing literature. The DC analysis was performed by first generating a Pearson correlation matrix between the time series of every voxel in a WM mask and all other brain voxels. To minimize the influence of spurious correlations, a threshold of *r* > 0.25 was applied. Finally, the voxel-wise DC values were converted to a z-score map using Fisher’s z-transformation to ensure normality for subsequent group-level analysis (34). Finally, the MNI coordinates were converted to IIT coordinates using the method posted by the IIT Atlas developers on the forums (https://www.nitrc.org/forum/forum.php?thread_id=4883&forum_id=1850) for comparison with the differences derived from the DTI analysis. Subsequently, group comparisons of ALFF and DC values were performed. For specific statistical methods, see Section 2.8.1.

### 2.8 Statistical analysis

#### 2.8.1 Group comparisons

Demographic and clinical data were analyzed using SPSS statistical analysis software (version 27.0; IBM Corp., Armonk, NY, USA). All variables were evaluated for outliers, data entry errors, and missing values. Pearson’s chi-squared test was used to check for sex differences. The non-parametric Mann–Whitney U-test was used to analyze between-group differences in clinical data between MWoA patients and HCs. For the FA, MD, RD, and AD values, we used non-parametric inference to robustly control for multiple comparisons. This was implemented using FSL randomization with 5,000 permutations. The multiple comparison was corrected by threshold-free cluster enhancement (TFCE) at a significance level of *p* of < 0.05 (35). A two-sample t-test was performed to compare the ALFF and DC values between MWoA patients and HCs. The result that remained after Gaussian random field (GRF correlation, voxel *p* < 0.01, cluster size > 40, cluster *p* < 0.05) was considered to be significant (36). Our multimodal analysis used distinct correction strategies tailored to each data type’s spatial characteristics. For the TBSS analysis of the sparse, linear WM skeleton, we used TFCE to sensitively detect elongated signals without an arbitrary cluster-forming threshold (37). For ALFF and DC analysis, we dealt with full-volume voxel-based data across the whole brain. The GRF method effectively controls the family-wise error rate at the voxel level under the assumption of a continuous random field, providing a robust framework (38). This differentiated correction strategy ensures that statistical inference sensitivity is optimally aligned with the inherent spatial characteristics of each imaging modality. For all group analyses of the neuroimaging data, age, sex, and average absolute motion were included as covariates.

#### 2.8.2 Correlation analysis

Spearman’s rank correlation coefficients were calculated to explore relationships between variables of interest (clinical and neuroimaging data), controlling for age, sex, and average absolute motion.

#### 2.8.3 Mediation analysis

Using SPSS macro PROCESS (Model 4) for exploratory mediation analysis (39), we examined the statistical associations among clinical characteristics, DC values, ALFF values, and WM integrity. This toolbox dissects total effects, direct effects, and mediation effects within a general linear modeling framework. Mediation effects were estimated using 5,000 bootstrap samples, with statistical significance determined if the 95% confidence intervals (CIs) excluded zero. Age, sex, and average absolute motion were controlled as covariates.

## 3 Results

### 3.1 Demographic and clinical characteristics

The study initially included 53 patients with MWoA and 52 HCs. Two participants in the MWoA group and one participant in the HCs group were excluded due to head movement greater than 2 mm during scanning and incomplete data conversion. Finally, 51 MWoA and 51 HCs were included in the neuroimaging analysis.

There were no significant differences found in age (*p* = 0.726) or sex (*p* = 0.477) between the MWoA and HCs groups. There were 49 MWoA patients who had not taken any medication, while 2 MWoA patients took ibuprofen sustained-release capsules (400 mg) for acute pain relief during headache attacks. Clinical and demographic data are presented in Table 1.

**Table 1:** Demographics and clinical characteristics of patients with MWoA and HCs.

Characteristics	MWoA patients	HCs	Statistics	Effect size (95%CI)	*p*-values
*n*	51	51	–	–	–
Age (years), median (P25, P75)	33.000 (26.000, 42.000)	25.000 (25.000, 45.000)	*Z* = −1.164	*r* = 0.115; HL, mean (95%CI) = 2.000 (−1.000, 6.000)	0.244
Sex (female/male)	38/13	41/10	$x^{2}=0.505$	φ = 0.070 (−0.128, 0.258)	0.477
Disease duration (months), median (P25, P75)	72.000 (42.000, 120.000)	–	–	–	–
Migraine frequency (times per month), median (P25, P75)	4.000 (3.000, 5.000)	–	–	–	–
Duration of each headache (hours), median (P25, P75)	7.500 (3.200, 12.500)	–	–	–	–
VAS score, median (P25, P75)	5.000 (4.250, 6.600)	–	–	–	–
Hit-6 score, median (P25, P75)	65.000 (59.000, 67.000)	–	–	–	–
MSQ score mean, role restrictive domain, median (P25, P75)	62.800 (51.430, 74.290)	–	–	–	–
MSQ score mean, role preventive domain, median (P25, P75)	80.000 (65.000, 85.000)	–	–	–	–
MSQ score mean, emotional domain, median (P25, P75)	80.000 (66.670, 93.330)	–	–	–	–

MWoA, migraine without aura; HCs, healthy controls; VAS, visual analog scale; HIT-6, the Headache Impact Test; MSQ, Migraine-Specific Quality-of-Life Questionnaire; CI, confidence interval; *r*, the effect size for the Mann–Whitney U-test; φ, Phi coefficient; HL, Hodges–Lehmann estimator; “–”: no data.

### 3.2 WM microstructural alterations in MWoA

**Figure 1:**
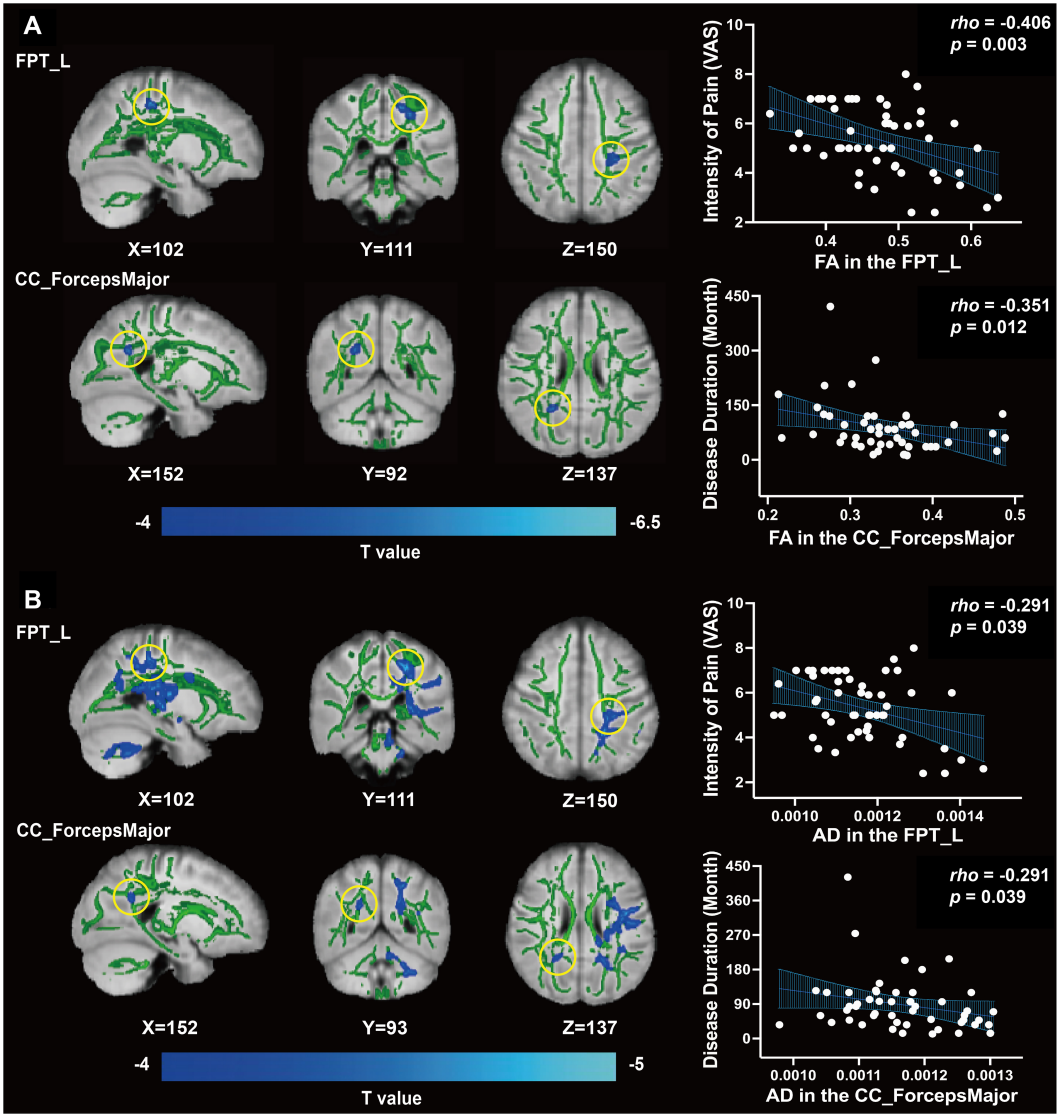
Differences of WM fiber tracts in DTI and clinical correlation. **(A)** The FA value of FPT_L, CC_ForcepsMajor, and clinical correlation (*p* < 0.05). **(B)** The AD value of FPT_L, CC_ForcepsMajor, and clinical correlation (*p* < 0.05).

FA (peak *t* = −4.150, TFCE corrected, *p* = 0.034, Table 2, Figure 1A) and AD (peak *t* = −4.881, TFCE-corrected, *p* < 0.01, Figure 1B) values were decreased in the left frontopontine tract (FPT_L) of patients with MWoA compared to HCs. There was also a decrease in FA (peak *t* = −6.244, TFCE corrected, *p* = 0.030, Table 2, Figure 1A) and AD (peak *t* = −4.231, TFCE corrected, *p* = 0.034, Table 2, Figure 1B) values in the forceps major (CC_ForcepsMajor) among patients with MWoA compared to HCs. No WM fiber tracts showed an increase in FA and AD values. However, TBSS analysis revealed no statistically significant differences in MD or RD between the MWoA and HC groups, as no clusters survived TFCE correction for multiple comparisons (*p* > 0.05).

**Table 2:** Differences of WM fiber tracts in DTI (TFCE-corrected).

WM fiber tracts	IIT coordinate			*p-*values	Peak *t*
	X	Y	Z		
FA values					
MWoA patients < HCs					
FPT_L	102	111	150	0.034	−4.150
CC_ForcepsMajor	152	92	137	0.030	−6.244
AD values					
MWoA patients < HCs					
FPT_L	102	111	150	0.005	−4.881
CC_ForcepsMajor	152	93	137	0.034	−4.231

WM, white matter; IIT, IIT Human Brain Atlas; MWoA, migraine without aura; HCs, healthy controls; FA, fractional anisotropy; FPT_L, left frontopontine tract; CC_ForcepsMajor, forceps major; AD, axial diffusivity.

Based on the abnormal coordinates, the most probable brain connections indicate that FPT_L links the left hemisphere postcentral cortex (ctx-lh-postcentral) with the left thalamus proper, while the CC_ForcepsMajor connects the right thalamus proper with the right hemisphere precuneus (ctx-rh-precuneus) (Table 3).

**Table 3:** The most probable brain connections for abnormal coordinates.

No	WM fiber tracts	IIT coordinate			Brain connections
		X	Y	Z	
1	FPT_L	102	111	150	ctx-lh-postcentral and left thalamus proper
2	CC_ForcepsMajor	152	92	137	Right thalamus proper and ctx-rh-precuneus

WM, white matter; IIT, IIT Human Brain Atlas; FPT_L, left frontopontine tract; CC_ForcepsMajor, forceps major; ctx, cortex; l h, left hemisphere; rh, right hemisphere.

We found that both FA and AD values of the FPT_L were inversely correlated with the score of VAS (*rho* = −0.406, *p* = 0.003, Figure 1A; *rho* = −0.291, *p* = 0.039, Figure 1B). Both FA and AD values of CC_ForcepsMajor were inversely correlated with disease duration (*rho* = −0.351, *p* = 0.012, Figure 1A; *rho* = −0.291, *p* = 0.039, Figure 1B). No significant correlations were found between the FA and AD values in the FPT_L or CC_ForcepsMajor and between migraine frequency, duration of each headache, VAS, HIT-6, or MSQ scores.

### 3.3 ALFF and DC alterations in MWoA

**Figure 2:**
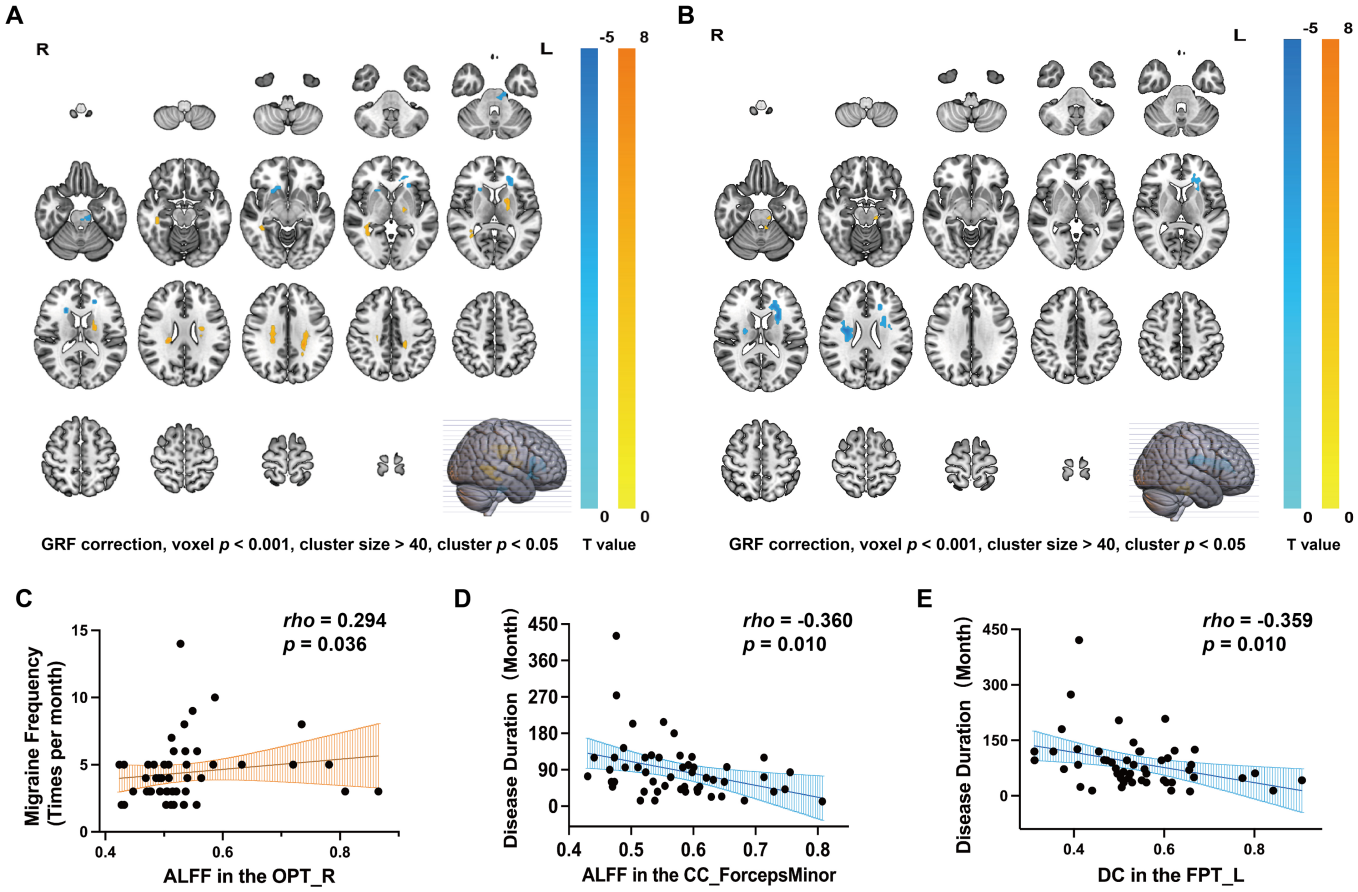
Differences of WM fiber tracts in ALFF, DC, and corresponding clinical characteristics. **(A)** ALFF changes in MWoA and corresponding clinical characteristics (GRF correction, voxel *p* < 0.001, cluster size > 40, cluster *p* < 0.05). **(B)** DC changes in MWoA and corresponding clinical characteristics (GRF correction, voxel *p* < 0.001, cluster size > 40, cluster *p* < 0.05). **(C)** The ALFF value of OPT_R was positively correlated with migraine frequency (*p* < 0.05). **(D)** The ALFF value of CC_ForcepsMinor was inversely correlated with disease duration (*p* < 0.05). **(E)** DC values for FPT_L were inversely correlated with disease duration (*p* < 0.05).

Compared to HCs, ALFF values among MWoA patients were increased in the FPT_L, CC, and the right occipitopontine tract (OPT_R) (GRF correction, voxel *p* < 0.001, cluster size > 40, cluster *p* < 0.05, Table 4, Figure 2A) and decreased in the forceps minor (CC_ForcepsMinor), the right frontal aslant tract (AST_R), and the left corticospinal tract (CST_L) (GRF correction, voxel *p* < 0.001, cluster size > 40, cluster *p* < 0.05, Table 4, Figure 2A). Compared to HCs, MWoA patients had increased DC values in the left spinothalamic tract (STT_L) (GRF correction, voxel *p* < 0.001, cluster size > 40, cluster *p* < 0.05, Table 4, Figure 2B) and decreased DC values in the FPT_L and AST_R (GRF correction, voxel *p* < 0.001, cluster size > 40, cluster *p* < 0.05, Table 4, Figure 2B).

**Table 4:** Differences of WM fiber tracts in ALFF and DC (GRF correction, voxel *p* < 0.001, cluster size > 40, cluster *p* < 0.05).

WM fiber tracts	Cluster size (voxels)	MNI coordinate			IIT coordinate			Peak *t*
		X	Y	Z	X	Y	Z	
ALFF values								
MWoA patients > HCs								
FPT_L	160	−24	−33	36	104	112	145	2.756
CC	82	21	−27	27	149	118	136	3.249
OPT_R	82	36	−45	3	164	100	112	3.063
MWoA patients < HCs								
CC_ForcepsMinor	66	−24	27	3	104	172	112	−2.937
AST_R	62	21	21	15	149	166	124	−2.944
CST_L	43	−9	−26	−27	119	119	82	−3.099
DC values								
MWoA patients > HCs								
STT_L	42	−12	−27	−21	116	118	88	4.237
MWoA patients < HCs								
FPT_L	194	−24	15	15	104	160	124	−5.217
AST_R	115	30	−6	24	158	139	133	−5.297

WM, white matter; MNI, Montreal Neurological Institute; IIT, IIT Human Brain Atlas; ALFF, amplitude of low-frequency fluctuation; MWoA, migraine without aura; HCs, healthy controls; FPT_L, left frontopontine tract; CC, corpus callosum; OPT_R, right occipitopontine tract; CC_ForcepsMinor, forceps minor; AST_R, right frontal aslant tract; CST_L, left corticospinal tract; DC, degree centrality; STT_L, left spinothalamic tract.

We found that the ALFF value of the OPT_R was positively correlated with migraine frequency (*rho* = 0.294, *p* = 0.036, Figure 2C), and the ALFF value of CC_ForcepsMinor was inversely correlated with disease duration (*rho* = −0.360, *p* = 0.010, Figure 2D). DC values of the FPT_L were inversely correlated with disease duration (*rho* = −0.359, *p* = 0.010, Figure 2E). No significant correlations were found between ALFF values, DC values, and clinical characteristics (disease duration, migraine frequency, duration of each headache, VAS, HIT-6, or MSQ scores) for the remaining differential WM fiber tracts.

**Table 5:** Mediation analysis of the total, direct, and indirect effects of DC in FPT_L on disease duration.

Type	Paths	B	SE	*t*	*p*-values	95% CI		β	Proportion mediated
						Lower	Upper		
Total effect	DC → disease duration	−193.369	77.242	−2.503	0.016	−348.761	−37.977	−0.338	–
Direct effect	DC → disease duration	−21.859	92.901	−0.235	0.815	−209.090	165.373	−0.038	–
Indirect effect	DC → ALFF→ disease duration	−39.605	26.146	–	–	−106.726	−4.804	−0.069	20.482%
	DC → FA → disease duration	−68.866	41.038	–	–	−167.697	−4.947	−0.120	35.612%
	DC → AD→ disease duration	−63.040	43.025	–	–	−165.002	−1.866	−0.110	32.595%
	Total	−171.511	68.292	–	–	−328.213	−59.963	−0.300	88.695%

DC, degree centrality value in the left frontopontine; ALFF, amplitude of low-frequency fluctuation value in the forceps minor; FA, fractional anisotropy value in the forceps major; AD, axial diffusivity in the forceps major; B, unstandardized coefficients; SE, standard error; CI, confidence interval; β, standardized coefficients.

### 3.4 The relationship between clinical characteristics and WM abnormalities in MWoA

**Figure 3:**
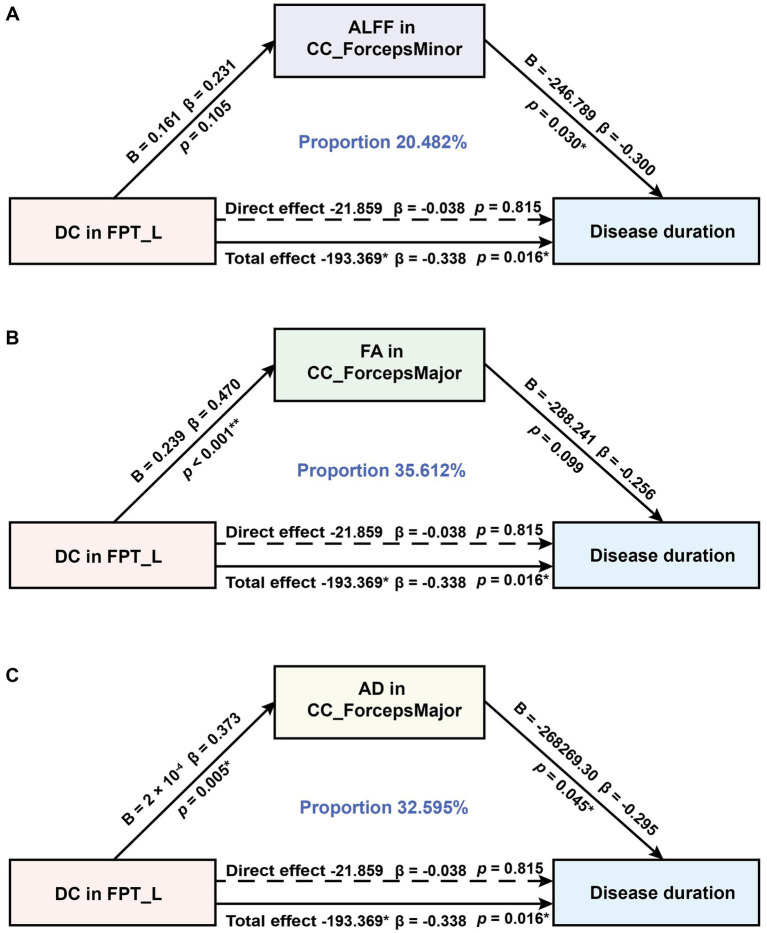
Mediation analysis to test whether ALFF, FA, and AD in CC subregions mediate the relationship between DC in FPT_L and disease duration in MWoA. **(A)** Results from the mediation effects of DC in FPT_L on disease duration through ALFF in the CC_ForcepsMinor. **(B)** Results from the mediation effects of DC in FPT_L on disease duration through FA in the CC_ForcepsMajor. **(C)** Results from the mediation effects of DC in the FPT_L on disease duration through AD in the CC_ForcepsMajor. B, unstandardized coefficients; *β*, standardized coefficients. *p < 0.05, **p < 0.01.

In MWoA, concurrent structural and functional abnormalities associated with disease duration were observed specifically in ALFF of the CC_ForcepsMinor, DC of the FPT_L, and FA and AD of the CC_ForcepsMajor. Further analysis revealed positive correlations between DC in the FPT_L and ALFF in the CC_ForcepsMinor (*rho* = -0.359, *p* = 0.010; *rho* = -0.360, *p* = 0.010), FA and AD in the CC_ForcepsMajor (*rho* = -0.351, *p* = 0.012; *rho* = -0.296, *p* = 0.039). We constructed an exploratory model with disease duration as the outcome variable. This model specification, which conceptualizes disease duration as a reference frame for progression, allows us to test if imaging metrics associate with this framework via CC abnormalities. It probes a biologically plausible mechanism, and within our cross-sectional design, these findings are exploratory and hypothesis-generating. According to the mediation analysis, the total effect of DC value on disease duration was statistically significant (total effect: *B* = −193.369, 95% CI [−348.761, −37.977], *β* = −0.338). The total indirect effect was also significant (total indirect effect: *B* = −171.511, 95% CI [−328.213, −59.963], *β* = −0.300, proportion mediated = 88.695%), while the direct effect was not significant (direct effect: *B* = −21.859, *β* = −0.038, 95% CI [−209.090, 165.373]). The mediation analysis identified three significant specific mediators: ALFF in the CC_ForcepsMinor (indirect effect: *B* = −39.605, 95% CI [−106.726, −4.804], *β* = −0.069, proportion mediated = 20.482%, Figure 3A), FA in the CC_ForcepsMajor (indirect effect: *B* = −68.866, 95% CI [−167.697, −4.947], *β* = −0.120, proportion mediated = 35.612%, Figure 3B), and AD in the CC_ForcepsMajor (indirect effect: *B* = −63.040, 95% CI [−165.002, −1.866], *β* = −0.110, proportion mediated = 32.595%, Figure 3C) (Table 5).

## 4 Discussion

This study is among the first to explore abnormal WM microstructure and function among MWoA patients by integrating DTI and rs-fMRI data. We observed that WM structural and functional abnormalities among MWoA patients were primarily located in the FPT_L and CC subregions, and these alterations were significantly correlated with clinical measures. Altered structural connectivity was observed between the right thalamus proper and the ctx-rh-precuneus, as well as between the ctx-lh-postcentral and the left thalamus proper. Our mediation analysis suggested that diminished functional integration in the FPT_L is associated with disease duration. Specifically, this association appears to operate through a pathway that involves both aberrant functional fluctuations and impaired microstructural integrity in CC subregions.

### 4.1 MWoA patients exhibit concurrent abnormalities in both WM structure and WM function

In patients with MWoA, we observed reduced FA and AD in the FPT_L and CC_ForcepsMajor, without significant changes in MD or RD. This pattern suggests impaired axonal integrity with relative myelin preservation. Notably, the severity of these microstructural abnormalities was inversely correlated with clinical pain intensity. Concurrently, the FPT_L exhibited a dissociation between increased ALFF and decreased DC. Recent studies on WM suggest that increased ALFF could reflect elevated local neural activity, which may be compensatory or dysregulated in nature, while reduced DC reflects impaired integration within broader brain networks (32, 33). Thus, the increased ALFF may be a failed compensatory response to the structural lesion, while the reduced DC directly manifests as the impaired coordination with key pain modulatory nodes, ultimately leading to heightened pain. However, the neurophysiological basis for the dissociation between ALFF and DC in WM remains unclear. Consequently, our interpretation should be considered a preliminary hypothesis requiring future validation. Methodologically, our analysis followed a structure-first approach, based on established paradigms from GM research (40–42). However, this method may overlook functional changes in regions that appear structurally intact. Future studies could mitigate this selection bias using independent whole-brain analyses for both modalities.

The WM abnormalities in migraine patients in previous studies were primarily located in the cerebellum and brainstem, CC, and thalamus, which differed from the results of our study (10, 11, 43). This discrepancy may be attributed to the fact that we focused on MWoA patients. In contrast, previous studies included various migraine subtypes, which may have distinct patterns of WM abnormalities. Despite this difference, a potential connection to the established role of the cerebellum and brainstem in migraine pathophysiology may still exist. FPT_L originates from the prefrontal cortex of the left frontal lobe and projects to the ipsilateral pontine nuclei. Clinical studies have identified the left dorsolateral prefrontal cortex (DLPFC) as a critical node in pain descending inhibition and a potential target for brain analgesia (44). A study suggests that structural and functional abnormalities in the frontal lobes may contribute to new daily persistent headache pathophysiology (45), which may be related to MWoA. The pontine nuclei are located in the brainstem and are responsible for relaying information from the cerebral cortex to the cerebellum. Previous studies have revealed that the pons is involved in pain modulation and that there exists a pontine inhibitory descending pathway (46). This suggests that the FPT_L, prefrontal cortex, cerebellum, and brainstem may collectively form an integrated pain-processing network. Specifically, the FPT_L may constitute the key WM substrate through which the prefrontal cortex regulates the pontine analgesia pathway. Consequently, the FPT_L, prefrontal cortex, and brainstem may function together as a system for top-down pain modulation. Microstructural damage and functional dysregulation of the FPT_L could disrupt this pathway, impairing top-down inhibitory control and thereby increasing pain intensity in patients with MWoA. These alterations may serve as potential imaging biomarkers to identify MWoA patients with impaired top-down pain modulation. Moreover, these findings provide a neuroanatomical basis for optimizing neuromodulation therapies. Prior research has established that high-frequency repetitive transcranial magnetic stimulation (rTMS) of the left DLPFC alleviates migraine pain (47). Currently, our findings reveal a potential mechanism for this therapy, indicating that the FPT_L serves as a key structural conduit linking the stimulation site to downstream pain modulatory centers. Consequently, future studies should investigate the FPT_L within the broader prefrontal-pontine-cerebellar network. This will clarify its role in pain modulation and guide the development of targeted therapeutics for MWoA.

The CC, which connects the cerebral hemispheres, is critical for interhemispheric integration and coordination (48). WM microstructural changes in CC_ForcepsMajor were inversely correlated with disease duration, which is consistent with several previous studies about CC (12, 49). This finding suggests that interhemispheric integration is compromised during the development of migraine (50), potentially related to neuroplastic adaptations in chronic pain. However, we detected no concomitant functional abnormalities in the CC_ForcepsMajor, which may imply the engagement of compensatory mechanisms to preserve functional connectivity despite structural decline. In contrast, decreased ALFF in the CC_ForcepsMinor was also inversely correlated with disease duration, highlighting this subregion’s potential vulnerability to functional disruption. Collectively, abnormalities in both the structure and function of CC subregions may impair interhemispheric integration, thereby dysregulating pain sensory processing in MWoA. Future studies should evaluate whether treatment-induced clinical improvements correlate with neuroplastic changes in the callosal WM. Establishing this link would pave the way for a more precise, mechanism-based paradigm in migraine management.

Furthermore, while extant research on the right frontal aslant tract (AST_R) has focused on its roles in executive function and language control (7, 51), our study revealed co-occurring reductions in ALFF and DC within this tract. This convergent functional decline suggests that the AST_R may represent a previously underrecognized pathological node in MWoA. This may indicate that functional decline in this fiber tract precedes damage to microstructure. Thus, AST_R may also be an underrecognized pathological fiber tract in MWoA.

### 4.2 Abnormal brain connectivity in MWoA patients

To map the connections of abnormal WM tracts, we leveraged the IIT atlas to visualize the most probable pathways for the relevant voxels. This approach, while not a direct validation, enabled reasonable functional inferences from the structural abnormalities. We identified two connections associated with differential WM fiber tracts in the CC_ForcepsMajor and FPT_L: the connection between the right thalamus proper and the ctx-rh-precuneus and the connection between the ctx-lh-postcentral and left thalamus proper. The thalamus serves as a relay station for pain signals and plays a crucial role in pain mechanisms (52, 53). Thus, the thalamus may integrate pain processing through these connections. Previous studies have shown that functional connectivity abnormalities in the thalamus of MWoA may exacerbate pain perception through thalamocortical interactions with the visual cortex (54, 55). Furthermore, studies have shown that the abnormalities of the precuneus were correlated with the clinical characteristics in MWoA (56, 57). In the present study, reduced FA and AD values of abnormal voxels in the right thalamus proper and ctx-rh-precuneus were inversely correlated with the duration of MWoA, suggesting that structural degradation of this connection may be associated with the length of disease duration. Reduced FA and AD values of abnormal voxels in the ctx-lh-postcentral and left thalamus proper were inversely correlated with headache intensity, suggesting a link between structural degradation of this connection and symptom severity. In summary, these connectivity abnormalities may constitute a critical network underpinning pain perception and disease progression in MWoA. This provides a valuable direction for future investigations into the structural network characteristics of abnormal WM fiber tracts in MWoA.

### 4.3 Association between functional integration and disease duration was mediated by functional fluctuations and integrity of WM and disease duration

Our mediation analysis revealed a significant total effect of DC values in the FPT_L on disease duration. However, 88.7% of this effect association was mediated through ALFF, FA, and AD in CC subregions, while the direct effect association was non-significant. This pattern suggests that the association between functional integration in the FPT_L and disease duration can be largely accounted for by functional fluctuations and WM microstructural integrity in the CC subregions. Furthermore, the FA pathway demonstrated the strongest mediation effect. This finding suggests that impaired WM integrity in CC subregions may represent a key potential pathway linking reduced functional integration to disease progression. Collectively, our findings support a statistical model in which reduced DC in the FPT_L is associated with longer disease duration, alongside co-occurring alterations in the CC functional and microstructural integrity. Together, these measures form a multimodal set of candidate biomarkers, offering a new perspective for understanding disease progression and identifying potential therapeutic targets in MWoA. Notably, the cross-sectional design of this study limits causal interpretation of the observed mediation effects. However, by constructing a directional model with disease duration as the outcome variable, we outline a statistically consistent associative pathway. These findings are primarily exploratory and aim to provide direction for subsequent longitudinal research.

Previous studies have suggested a link between the structure and function of the GM in MWoA patients (58, 59), yet the relationship between the microstructure of the WM and its functional and clinical characteristics has been largely overlooked. By using mediation analysis, we extend previous findings of a negative correlation between CC microstructure and disease duration (12, 49). This approach offers a preliminary model of their interaction, thereby enhancing our understanding of complex neural mechanisms underlying MWoA.

### 4.4 Limitations

Several limitations should be acknowledged. (1) Due to the cross-sectional nature of our study, we can demonstrate an association but cannot establish a causal relationship between the observed WM abnormalities and clinical symptoms. Future longitudinal studies are necessary to confirm the temporal sequence of these changes. (2) The sample size was determined based on conventions from previous similar studies (60–62) rather than an *a priori* power analysis, which may have resulted in insufficient power to detect subtle effects. (3) The small sample size of the present study, due to the restricted conditions, may limit the generalizability of the results, and a multicenter, large sample study is needed to validate the reproducibility of the findings.

## 5 Conclusion

This study demonstrates that WM fiber tracts (the FPT_L and CC subregions) with co-occurring structural and functional abnormalities may be associated with pain intensity and disease duration. Furthermore, our results suggest that aberrant functional fluctuations and impaired WM integrity may serve as mediating factors in the relationship between reduced functional integration and disease progression. These results provide further evidence of the WM abnormality hypothesis of MWoA and provide new perspectives for future exploration of MWoA interventions targeting WM.

## Data Availability

The raw data supporting the conclusions of this article will be made available by the authors, without undue reservation.
